# Clinical Outcome and Wound Healing following Carpal Tunnel Decompression: A Comparison of Two Common Suture Materials

**DOI:** 10.1155/2014/270137

**Published:** 2014-08-07

**Authors:** Robert J. MacFarlane, Thomas D. Donnelly, Yousaf Khan, Syam Morapudi, Mohammad Waseem, Jochen Fischer

**Affiliations:** Department of Trauma and Orthopaedics, Macclesfield District General Hospital, East Cheshire NHS Trust, Victoria Road, Macclesfield SK10 3BL, UK

## Abstract

*Introduction*. Debate exists amongst surgeons regarding the ideal suture material for skin closure in carpal tunnel decompression (CTD). This study compares wound related complications, patient satisfaction, and functional outcome following open carpal tunnel decompression in patients undergoing wound closure with either of two common absorbable and nonabsorbable suture types. *Materials and Methods*. 53 patients underwent CTD with either 4/0 polypropylene (ProleneTM, *n* = 28) or 4/0 polyglactin (Vicryl RapideTM, *n* = 25) for skin closure. QuickDASH, VAS satisfaction scores, and Southampton wound scores were assessed preoperatively and at 2 and 6 weeks postoperatively. *Results*. At 6 weeks the mean QuickDASH scores postoperatively were 18.54 and 17.70 for absorbable and nonabsorbable sutures, respectively, (*P* = 0.86). The mean VAS scores were 0.61 and 0.42 (*P* = 0.91), respectively. All patients achieved a Southampton wound score of 0 by 6 weeks except one, who achieved 1C in the nonabsorbable group, equivalent to mild erythema. There were no complications in either group. *Conclusion*. Both suture types are safe and effective materials for CTD, and we recommend surgeons to choose according to personal preference, handling properties, and resources available for suture removal.

## 1. Introduction

Carpal tunnel syndrome (CTS) is a common condition affecting approximately 3.8% of the UK population [1]. It can cause considerable functional disability and patient morbidity, with the median number of resultant days away from work annually due to CTS as high as 27 days [2]. CTS commonly presents with pain, sensory disturbance, and paraesthesia in the distribution of the median nerve in the affected hand. Treatment involves analgesia, splinting, injections, or surgical division of the flexor retinaculum. Traditionally carpal tunnel decompression (CTD) has been performed by an open procedure with good results stretching back many years [3, 4]. Endoscopic carpal tunnel release (originally performed in 1987 [5]) has also achieved positive results [6, 7] but is associated with a number of complications and requires more expensive materials. Many surgeons prefer open release due to the shorter operating time, lower equipment requirements, and cost [8].

The use of absorbable sutures is becoming more frequent in hand surgery, as they do not require removal, which may be painful or unpleasant for the patient, and do not require an additional appointment at a suitable postoperative date for removal. Absorbable sutures have been associated with an immunogenic response during the postoperative period which can lead to wound healing problems such as sterile suture abscess and granuloma formation [9]. Specifically, this inflammatory response is a local foreign body reaction, with infiltration of macrophages responding to proinflammatory cytokines, and the subsequent formation of giant cells [10, 11]. There is also evidence that absorbable sutures result in higher residual wound inflammation in comparison to nonabsorbable suture materials [12, 13]. Many surgeons therefore prefer to use absorbable suture materials still.

It was noted in our unit that a range of responses were given by patients postoperatively following open carpal tunnel release using the two suture materials studied. Moreover, patient satisfaction following upper limb surgery is related to both clinical and functional outcomes, and, in addition, cosmetic result [16]. The principal aim of our study was therefore to compare two common suture types used in open CTD, including the most commonly used absorbable and nonabsorbable suture in our institution. Specifically, we aimed to examine functional outcome following surgery, patient satisfaction, in terms of both pain and cosmetic appearance, at 2 and 6 weeks postoperatively.

## 2. Materials and Methods

We performed a prospective comparative study over 5 months of all patients listed for primary carpal tunnel decompression, from the elective hand surgery outpatient clinic at Macclesfield General Hospital, UK, between 13/03/2010 and 27/07/2010.

Patients with a suggestive history and clinical signs were sent for neurophysiological assessment. Those with a diagnosis of carpal tunnel syndrome confirmed both clinically and neurophysiologically were offered open decompression of the carpal tunnel under local anaesthesia. Inclusion criteria for the study were adult patients, with primary CTS, confirmed neurophysiologically. Exclusion criteria were previous carpal tunnel surgery, other previous palmar surgeries with local scarring, other pathologies affecting the hand, for example, Dupuytren's disease, systemic pathology with peripheral manifestations, for example, psoriasis, rheumatoid arthritis, skin conditions which may otherwise affect wound healing, known allergy to suture materials, previous keloid or hypertrophic scarring, or concurrent steroid or chemotherapy treatment.

**Figure 1:**
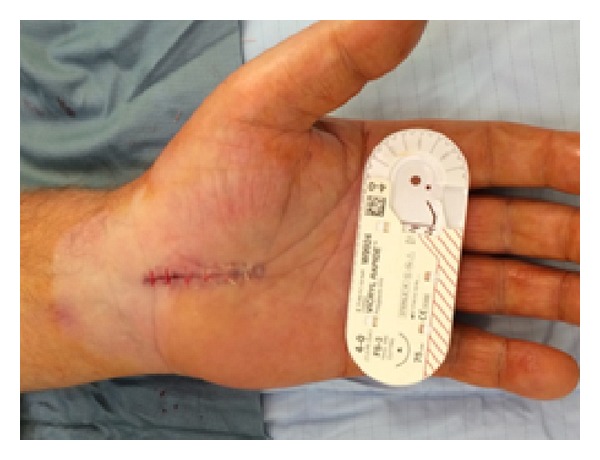
Carpal tunnel wound after closure with Vicryl Rapide.

**Figure 2:**
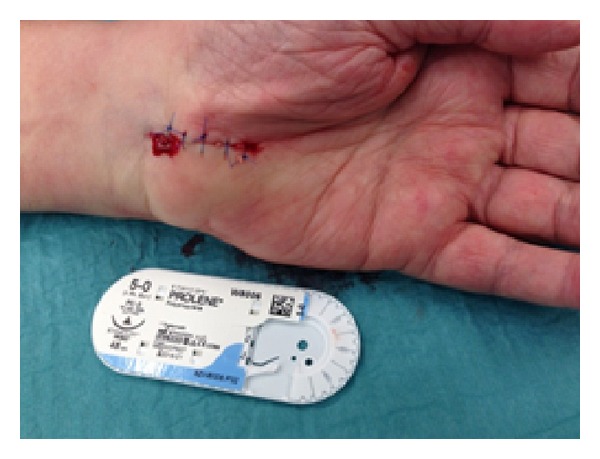
Carpal tunnel wound after closure with Prolene.

All procedures were performed under local anaesthesia and under tourniquet control, using 1% lidocaine solution infiltrated into the palmar soft tissues and the carpal tunnel itself. All cases were performed by either of the two senior authors (MW or JF) with one using an absorbable suture (4/0 Vicryl Rapide, Ethicon, UK, see Figure 1) and the other a nonabsorbable suture. (5/0 Prolene, Ethicon, UK, see Figure 2). Incisions were made from the distal wrist flexor crease in line with the radial border of the ring finger, approximately 3 cm in length up to the level of the thumb metacarpophalangeal joint. Wound closure was with interrupted mattress sutures with either of the materials being studied. Sterile dressing was applied for 10 days with crepe bandage for the first 48 hours.

**Figure 3:**
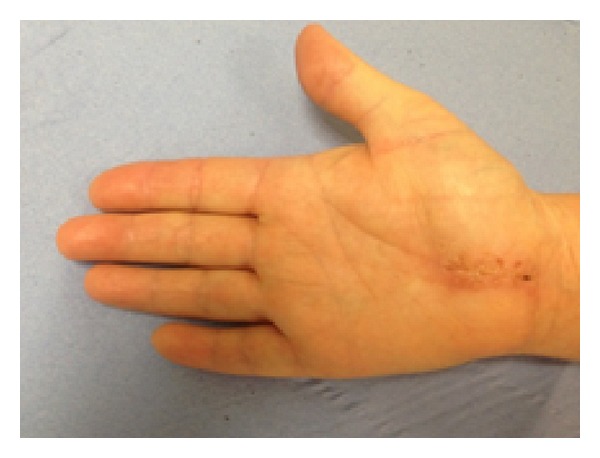
Typical appearance of a healed carpal tunnel wound at 6 weeks postoperatively. The suture material used in this case was Vicryl Rapide.

Patients were assessed preoperatively with Quick Disabilities of Arm Shoulder and Hand functional score (QuickDASH) and visual analogue (VAS) pain scores. Patients underwent suture removal and wound inspection in the outpatient clinic at 14 days postoperatively, with wound appearance and any notable findings recorded in the patient notes, including a Southampton wound score. Patients returned to clinic at six weeks postoperatively for clinical review with a visual analogue scale (VAS) pain and satisfaction scores relating specifically to the scar, and further QuickDASH scores were recorded (see Figure 3). Data were compiled and analysed using Microsoft Excel, and data between absorbable and nonabsorbable groups were compared using unpaired Student's *t*-test and chi-squared test.

## 3. Results

A total of 53 patients were recruited to the study; 23 were male and 30 female. The mean age was 58.6 (29.5–84.8) for the absorbable (*n* = 25) and 56.7 (19.5–81.5) for the nonabsorbable group (*n* = 28); *P* value = 0.69 (Table 1).

At 2 weeks 16/25 pts in the absorbable group had achieved grade 1 (normal healing with mild bruising or erythema) and 9/25 had achieved grade 2 (erythema with other signs of inflammation). In the nonabsorbable group, 19/28 had achieved grade 1 whilst 9/28 had achieved grade 2 (*P* = 0.6). At 6 weeks, all patients in the absorbable group had achieved a score of 0 (normal healing), and all but 1 patient in the nonabsorbable group had achieved a score of 0. One patient, from the nonabsorbable cohort, had a Southampton wound score of 1c at six weeks, indicating normal healing but with surrounding erythema, which subsequently settled spontaneously. There were no wound infections in either group at the two or six weeks intervals. No other complications were recorded.

**Figure 4:**
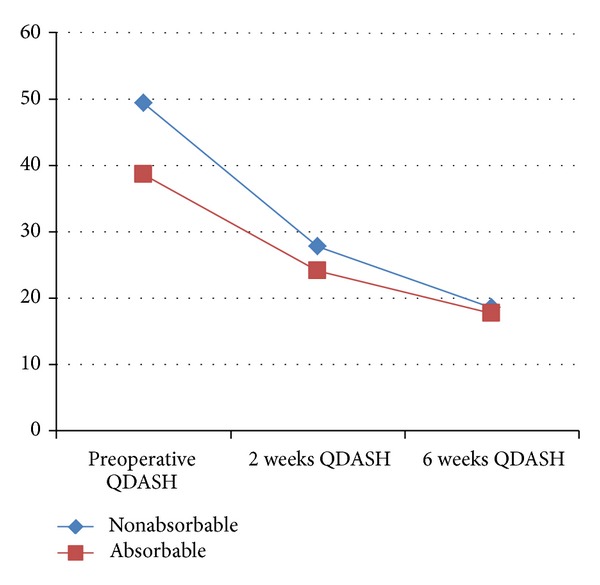
Functional outcome using QuickDASH.

The mean preoperative QuickDASH score was 49.39 (range 12.5–79.55) in the absorbable group and 38.63 (13.63–86.36) in the nonabsorbable group. At 6 weeks mean QuickDASH was 18.54 (range 0.00–63.64, SD 17.43) in the absorbable group and 17.70 (range 2.27–40.91, SD 11.85) in the nonabsorbable group (*P* = 0.5, see Figure 4). Mean wound length was 3.34 cm in the absorbable group (range 2.0–4.5, SD 0.54) and 3.95 in the nonabsorbable group (range 3.0–5.0, SD 0.74), and this difference reached significance (*P* = 0.03). The mean VAS patient satisfaction scores at 6 weeks after surgery were 0.61 in the absorbable group (range 0–4, SD 1.46) and 0.42 in the nonabsorbable group (range 0–3, SD 1.02, *P* = 0.6) indicating no difference between the groups in relation to satisfaction with wound appearance or symptoms (see Table 2).

## 4. Discussion

A number of authors have compared the use of absorbable and nonabsorbable sutures in carpal tunnel surgery (see Table 3). Erel et al. evaluated 64 patients receiving either continuous subcuticular polyglactin (Vicryl) or interrupted Prolene sutures, noting significantly greater VAS pain scores at 10 days postoperatively in the nonabsorbable group but no differences at 6 weeks in terms of healing, complications, or pain scores [12]. In a prospective randomized study of patients undergoing carpal tunnel release with either Vicryl Rapide or Novafil. Theopold et al. [14] observed 47 patients undergoing closure of open carpal tunnel release surgery using either 4/0 Vicryl Rapide or 4/0 Novafil (Covidien, Dublin, Ireland). Using the patient and observer scar assessment scale (POSAS) and numeric analogue scores for satisfaction, there were no differences in wound appearance, pain, or satisfaction at 2 or 6 weeks postoperatively. In a larger study, Menovsky et al. [17] prospectively studied 61 patients divided into three groups depending on method of skin closure [12, 15]. The authors used interrupted Nylon, subcuticular polyglactin (Vicryl), or stainless steel sutures. There were no differences in pain scores between the two groups at 2 or 6 weeks but noted an increased rate of complications, including wound infection and suture granulomata in the polyglactin group, recommending the use of nonabsorbable sutures for carpal tunnel surgery.

In the present study we have seen no difference in complications using the 2 suture types, which may reflect the faster dissolving Vicryl Rapide suture used in our study, which, in our experience, handles well and does not commonly result in suture granulomata. Furthermore, the use of interrupted sutures allows for knots to be placed in the outside of the skin which may come away from the wound when cleaning it at 2 weeks or simply can be trimmed away, without the risk of delayed absorption under the skin, which is sometimes seen with subcutaneous knot placement in continuous suture techniques.

In relation to hand surgery in general, comparative data have demonstrated variable outcomes between nonabsorbable and absorbable sutures. In a prospective, randomised study of 100 patients undergoing any elective hand or wrist surgery, Khundra et al. noted no significant difference between absorbable and nonabsorbable sutures (3/0 Vicryl Rapide and 3-0 Nylon) in terms of the cosmetic or functional results at 6 weeks and no difference in VAS score for satisfaction. These results support the findings in the present study, indicating that Vicryl Rapide appears to be a safe and effective absorbable suture in hand surgery and, more specifically, in open carpal tunnel release.

It is worth noting that some evidence has shown that absorbable sutures may cause an inflammatory response leading to persistent scar tenderness, erythema, and occasionally hypertrophic scar [9, 18]. However, nonabsorbable sutures require removal postoperatively, causing a degree of discomfort, and may also leave suture marks on the skin [18]. Furthermore, nonabsorbable sutures have been associated with significantly greater amounts of time spent on wound care, compared with absorbable sutures, during the first postoperative clinic appointment, with no comparable difference in pain scores or complication rates [19].

It is important to note that this study examined the use of Prolene and Vicryl Rapide, which have not previously been directly compared by other authors when used in carpal tunnel surgery. We have found little difference in outcome between the two products examined. Both the absorbable and the nonabsorbable suture materials had similar, positive, results regarding functional outcome, wound healing, and aesthetics in both groups. Furthermore, our results are comparable to those reported in the recent literature.

We acknowledge a number of clear limitations to this study. The groups were relatively small, and larger groups may have demonstrated a rate of complications or difference in outcome which was not seen in our data. Patients were not fully randomised which has resulted in the loss of some objectivity of the data. The short followup time of 6 weeks, although short, is similar to other studies examining suture materials in hand surgery.

This study is the largest in the literature comparing Vicryl Rapide and Prolene in the closure of open carpal tunnel release surgery in terms of both wound assessment and functional outcome. We would recommend that a larger, prospective randomised controlled trial will be conducted to examine longer term outcomes and observe complication rates in more detail.

## 5. Conclusions

We have found both materials used in this study to be safe and effective products for wound closure in carpal tunnel surgery. We recommend that choice of suture material is made based on surgeon preference and on local resources and infrastructure available for suture removal.

## Figures and Tables

**Table 1:** Demographics.

	Absorbable	Nonabsorbable	*P* value
Patients (M : F)	*n* = 25 (7 : 18)	*n* = 28 (16 : 12)	
Mean age (range)	58.6 (29.5–84.8)	56.7 (19.5–81.5)	0.69
Hand dominance (dominant : nondominant)	15 : 10	16 : 12	

**Table 2:** Clinical and functional outcomes.

	Absorbable	Nonabsorbable	*P* value
Mean wound length (range)	3.34 (2.0–4.5 cm)	3.95 (3.0–5.0)	0.003
Preoperative QuickDASH	49.39 (12.5–79.55)	38.63 (13.63–86.36)	
2 weeks QuickDASH	27.80 (20.33–35.17)	24.10 (4.55–65.90)	0.49
6 weeks QuickDASH	18.54 (0.00–63.64)	17.70 (2.27–40.91)	0.86
VAS score mean (SD)	0.61 (1.46)	0.42 (1.02)	0.91

**Table 3:** Summary of literature relating to suture materials in carpal tunnel surgery.

Author	Number of patients	Materials	Outcome
Present study (2013)	53	Vicryl Rapide and Prolene	No difference at 2 or 6 weeks comparing VAS, QuickDASH, or Southampton wound score

Erel et al. (2001) [12]	64	Vicryl and Prolene	Significantly greater VAS pain scores at 10 days in Vicryl but no difference at 6 weeks including wound healing and complication rates

Theopold et al. (2012) [14]	47	Vicryl Rapide and Novafil	No difference in POSAS and numeric scores assessing wound appearance, pain, or satisfaction at 2 and 6 weeks after procedure

Menovsky et al. (2004) [15]	61	Nylon, Vicryl (subcuticular), and stainless steel	No differences in pain scores at 2 and 6 weeks. Increased number of complications with Vicryl
